# Data augmentation-assisted deep learning of hand-drawn partially colored sketches for visual search

**DOI:** 10.1371/journal.pone.0183838

**Published:** 2017-08-31

**Authors:** Jamil Ahmad, Khan Muhammad, Sung Wook Baik

**Affiliations:** Department of Software, College of Software and Convergence Technology, Sejong University, Seoul, Republic of Korea; University College London, UNITED KINGDOM

## Abstract

In recent years, image databases are growing at exponential rates, making their management, indexing, and retrieval, very challenging. Typical image retrieval systems rely on sample images as queries. However, in the absence of sample query images, hand-drawn sketches are also used. The recent adoption of touch screen input devices makes it very convenient to quickly draw shaded sketches of objects to be used for querying image databases. This paper presents a mechanism to provide access to visual information based on users’ hand-drawn partially colored sketches using touch screen devices. A key challenge for sketch-based image retrieval systems is to cope with the inherent ambiguity in sketches due to the lack of colors, textures, shading, and drawing imperfections. To cope with these issues, we propose to fine-tune a deep convolutional neural network (CNN) using augmented dataset to extract features from partially colored hand-drawn sketches for query specification in a sketch-based image retrieval framework. The large augmented dataset contains natural images, edge maps, hand-drawn sketches, de-colorized, and de-texturized images which allow CNN to effectively model visual contents presented to it in a variety of forms. The deep features extracted from CNN allow retrieval of images using both sketches and full color images as queries. We also evaluated the role of partial coloring or shading in sketches to improve the retrieval performance. The proposed method is tested on two large datasets for sketch recognition and sketch-based image retrieval and achieved better classification and retrieval performance than many existing methods.

## Introduction

With the widespread use and adaptation of portable smart devices like phones and tablets in our day-to-day computing activities, sketch-based image retrieval (SBIR) has shown promising potential as an intuitive means to retrieve multimedia contents. The touch screen interface of these devices allow users to quickly and conveniently draw rough sketches of objects or scenes with their fingers and retrieve similar images from the collection of images contained in the repositories [1]. Hand drawn sketches are abstract representations of objects and scenes with imperfections in contours and noise. They differ greatly from black and white or full-color images, and poses several challenges in distinctive and robust representation for sketch based image retrieval applications [2]. In case the features of hand-drawn sketches are extracted and represented appropriately, they can serve as an effective means to specify queries when example images are unavailable.

Hand-drawn sketches are merely rough descriptions of scenes and objects and do not need to be artistic [3,4]. They are mainly composed of simple lines and strokes without any fill colors or details. These contours are considered as highly informative according to human perspective, and usually suffice for recognition by humans. In traditional sketch based retrieval systems, users need to fill sketches with colors to make them visually similar to full-color images. Though such techniques were considered as cumbersome for the users, the modern touch screen interfaces can make the process far more convenient than the traditional keyboard and mouse interfaces of the past.

Image retrieval systems must understand users’ intent while processing their queries. This is a difficult task which becomes even more severe in case of sketch-based queries due to inherent ambiguity caused by the absence of semantic information, textures, colors, and luminance. This ambiguity has been resolved previously by posing SBIR as model fitting approach attempted to align sketches with image data. However, such approaches carried with it huge computational costs. Other approaches attempted to extract local or global features from sketches and compared them with the features extracted from the edge maps of database images. In these approaches, content matching of images and sketches is accomplished using contour matching. In majority of these systems, the authors have used hand-engineered features like the variants of histogram of oriented gradients (HoG) [5], bag-of-visual words (BoVW) [6,7], and various local and global feature similarity methods. However, both local and global matching approaches have their shortcomings. For instance, the global contour matching approaches have to take into account the imprecise nature of hand-drawn sketches, thereby requiring some degree of tolerance. This approach in matching images with sketches often does not reflect content similarity. Though this problem has been reduced using local approaches, they are computationally very expensive. Several researchers attempted to address this issue by introducing efficient methods for reducing computational cost by sacrificing retrieval performance such as Wang et al. [2] who introduced an edgel index structure to efficiently solve sketch retrieval problem. However, their method heavily relied on local features, and the matching process was not very robust. Qian et al. [8] proposed a re-ranking and relevance feedback approach to address this issue which attempts to refine search results using relevance feedback mechanisms. Although such methods improve retrieval results based on local features, their retrieval performance still depends on the hand-engineered features which inherently lack capability to describe high level semantics in images.

To overcome these issues posed by hand-crafted local features based matching schemes in image retrieval systems, researchers have also used the recent powerful deep learning based approaches which are capable of modeling high level characteristics in images. The major advantage of these methods is that they can automatically learn features without requiring us to design algorithms for them. The recent advancements in image recognition due to these methods have motivated researchers to design powerful models to perform a variety of tasks. The authors in [9] proposed a sketch based image retrieval method using Siamese convolutional neural network (CNN). Their main idea was to derive similar features for image-sketch pairs that are marked relevant and derive dissimilar features for irrelevant ones. It was achieved by tuning two identical CNNs linked by one loss function. One of the CNN was tuned on the edge maps derived from full color images and the other on corresponding sketches. The joint output generated by the two linked models correspond to the degree of similarity between the two inputs. This way, images were matched with their corresponding sketches during retrieval phase by propagating sketch through one CNN and the image through the other. Though CNNs are known to be capable of learning high level representations in images and even edge maps, ignoring the color and texture aspects of images affect the overall representation process. Instead of eliminating essential aspect of visual media, i.e. color and texture, from the image matching process, we propose to optimize the inputs to allow learning of better representations with discriminative and deep CNN architectures. In this regard, we experimented with different data augmentation methods to allow effective representations of images that will also facilitate accurate matching with partially colored sketches. Though in the past, fully colored sketches were regarded as burdensome for the users, the current work targets portable smart devices where users can easily sketch and apply partial colors to various regions using onscreen tools on the touch screen devices. The objective of our work is to assess the suitability of deep CNNs for representing various facets of images including edge maps, de-texturized, edge enhanced, and de-colorized versions, so that optimal sketch based image retrieval system can be designed. We will also attempt to assess how the discriminative capabilities offered by the powerful deep CNNs are enhanced when these different representations of the same images are presented to them. Major contributions in this work are as follows:

1. Assess the representation capability of deep CNNs for hand-drawn sketches
2. Attempt to enhance sketch recognition using fine-tuning with augmented data
3. Evaluate the effects of data augmentation for sketch recognition and SBIR
4. Propose an optimal method for drawing partially colored sketches on portable smart devices
5. Determine optimal features from the fine-tuned CNN for sketch based retrieval

The rest of the paper is organized as: Section 2 presents a brief survey of state-of-the-art SBIR methods based on traditional hand-engineered features and deep features. The proposed method is illustrated in Section 3 and evaluated on two large datasets in Section 4. The paper concludes in Section 5 with strengths and weaknesses of the proposed method along with future directions.

## Related work

With the popularity of portable touch screen devices, people prefer to draw or write on touch screens instead of using pen. Touch screen devices make it very convenient to draw sketches and transform them into colorful drawings very quickly using onscreen controls. In the context of visual search in educational environments, sketch based image retrieval can make it more convenient to specify the query instead of textual query or looking for a sample image. Users can quickly draw a rough sketch of what they need and the retrieval engine will attempt to find relevant images or sketches from the dataset. Previous works on SBIR can be grouped into two categories based on the type of features they used to represent sketches.

### 2.1 Traditional approaches

A general workflow of a traditional SBIR system works by extracting edges from natural images in order to make them look like sketches and then extract hand-engineered features from the edge maps of images. The features of hand-drawn sketches are then matched with the features of edge maps to determine their similarity. Such methods are generally categorized into local and global approaches depending on how these features are extracted. In [10], authors extracted holistic features from sketches using edge pixels similar to shape context representation. Shao et al. [11] used similar features of sampled strokes to account for tolerate differences between sketches. Similarly, Cao et al. [12] developed an edge descriptor for facilitating sketch based image search. The main problem with these global representation schemes is that they are less effective in matching complex sketches. On the other hand local methods are more robust in representation. Elitz et al. [7] leveraged scale invariant features transform (SIFT) [13] to formulate bag-of-visual-words (BoVW) for SBIR. A similar approach based on the BoVW framework using histograms of gradients was presented for SBIR by Hu et al. in [5]. Both of these methods used k-means to build their corresponding codebooks. Xiao et al. [6] develop a method to extract shape words from sketches, followed by matching through Chamfer matching technique to perform shape matching. Shape words is a small segment of the sketch containing a group of connected edge pixels forming line segments and arcs. Each shape word have their own properties like direction, location, and size. Zhang et al. [14] further improved the shape words method by first discovering discriminative patches for various sketch categories. The shape patches are extracted from multiple scales, followed by construction of pyramid histogram. The discovery of discriminative patches is accomplished through an iterative procedure involving discriminative ranking and cluster merging. Major problems with these techniques is inherent complexity in matching boundaries of real images to roughly drawn sketches due to ambiguity, and imperfection. Furthermore, the semantic gap in hand-crafted image features causes SBIR method to significantly underperform in large datasets.

### 2.2 Deep learning based approaches

Deep CNNs have exhibited strong performance in a variety of computer vision tasks including image retrieval [15,16] and classification [17]. These methods have significantly outperformed traditional methods in so many other applications also. CNNs have the capability to automatically learn important features for a particular classification problem directly from the raw data (i.e. images). CNNs consist of several layers where each layer learns some characteristic of the data that can be used to perform the intended classification. Layers closer to the input learn low-level generic features, whereas higher layers in the network learn more complex features of the data, describing semantics and are considered higher level features. Babenko et al. [15] recently investigated features from the various layers of a trained CNN model for image retrieval. They showed that features extracted by a CNN (i.e. neural codes) are more discriminative and robust than the traditional hand-crafted features. To accomplish SBIR, Qi et al. [9] trained a Siamese CNN to map hand-drawn sketches to the edge maps of their corresponding images. Their framework consisted of two identical CNNs whose loss function was linked together. The sketch and edge map of the relevant image were forward propagated through the corresponding models, which attempted to decrease the feature distance between relevant pairs and increased the differences between irrelevant pairs. Their CNN consisted to three convolutional layers, each followed by a max pooling layers, and one fully connected layer. The output of the fully connected layer was input to the Softmax classifier. They showed superior retrieval performance than several state-of-the-art methods. However, they used a relatively simpler model and ignored color and texture features of the images while performing image matching. Wang et al. [18] presented a technique to train a CNN by mixing images as well as their edge maps or sketches to construct the training dataset. This enlarged augmented dataset consisting of both natural images as well as their sketches was used to train the CNN. The network they used consisted of five convolutional layers and three fully connected layers. During the training phase, they presented the network with 18 rotated versions of the sketch/edge map to further enhance discriminative ability of the network. During test phase, they created the 18 rotations of the query sketch and predicted the label by averaging output of the Softmax layer.

Deep CNNs are powerful architectures capable of yielding state-of-the-art performance in a variety of tasks. Their performance is limited by the availability of data which is usually solved with data augmentation techniques. The majority of these techniques used for SBIR either ignored color and texture features while representing images to be matched with simple sketches, or used data augmentation on a relatively smaller scale. However, we believe, that the touch screen devices make it far more convenient to draw partially colored sketches due to its ease of use. In such a setting, it can be more beneficial to use features of the full color images for searching relevant content to a partially colored sketch without requiring us to perform relevance feedback from the users. The efforts that we demand from users in refining the search results can be requested before entering the query. This can make the whole framework efficient and convenient.

## Visual search using partially colored sketches

**Fig 1:**
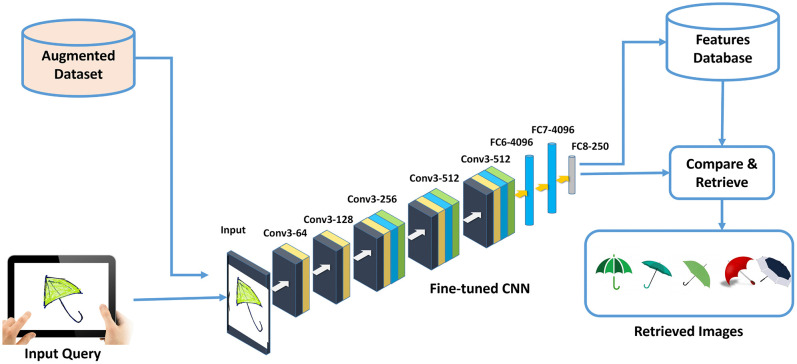
Schematic diagram of the proposed framework for SBIR. Umbrella image in Input Query and Input panels is an original image created by the authors. Umbrella images in Retrieved Images panel obtained from http://www.publicdomainpictures.net and https://publicdomainvectors.org/, available under a CC0 license.

This section presents the schematics of proposed framework including data augmentation, architecture of the deep CNN and its training, features extraction for sketch representation, and their retrieval. An abstract representation of the proposed framework is provided in Fig 1.

### 3.1 Data augmentation

**Fig 2:**
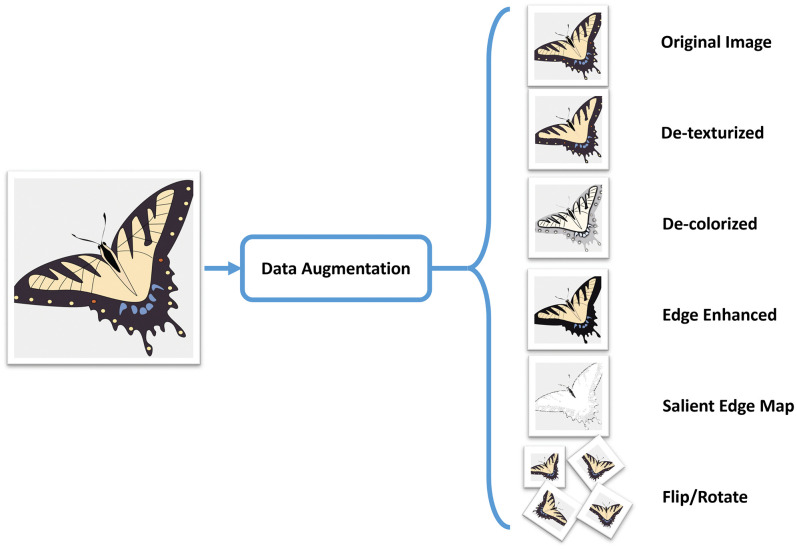
Data augmentation using semantic-preserving transformation for SBIR. Butterfly image reprinted and adapted from https://publicdomainvectors.org, available under a CC0 license.

Learning effectiveness of the deep CNNs are known to depend on the availability of sufficiently large training data. Data augmentation is an effective method to expand the training data by applying transformations and deformations to the labeled data, resulting in new samples as additional training data. A key attribute of the data augmentation is that the labels remain unchanged after applying those transformations. Generally, data augmentation involves crops, rotations, translations, scaling, and mirroring, etc. of the labeled samples. It has been shown that augmenting data during training phase improves the discriminative and generalization ability of the model [17]. In the context of SBIR, data augmentation has been used by Wang et al. [18] who expanded their training data by mixing sketches with real images. It allowed the CNN to learn features of the sketches in addition to features of the full image. We propose to use a more advanced method to augment training data by applying more transformations aimed at allowing CNN to robustly recognize partially colored sketches. Training data is augmented by mixing color images with salient edge maps, de-texturized, and de-colorized images obtained through anisotropic diffusion as shown in Fig 2. De-colorized and de-texturized versions of the images will allow them to be matched with partially shaded sketches. Similarly, the edge maps, hand-drawn sketches, and full color images will enable the CNN to effectively compare partially shaded sketches with full color images. The addition of these varying versions of images will enable CNN to model discriminative characteristics pertaining to these variety of representations. Furthermore, it will enable users to query the database using both natural images and sketches. We believe that training CNN with the augmented data will improve its generalization on unseen samples.

The decolorized images are obtained by transforming full color images into grayscale. Though this transformation can be obtained using a variety of methods, we opted to use the weighted conversion from RGB to grayscale using Eq (1).


$$I_{Gray}=0.299\times I_{R}+0.589\times I_{G}+0.112\times I_{B}$$ (1)

where I_R_, I_G_, and I_B_ are the red, green, and blue color channels respectively. The decolorized images tend to serve the purpose of representing shaded versions of images to the CNN during training. In a similar manner, the de-texturized images were formed by smoothing out fine textural content using anisotropic diffusion approach [19]. Salient edges are most likely contained in the hand-drawn sketch of any object. For allowing the CNN to model salient edges in images, we presented to it, the edge enhanced versions of images as well. These images were obtained by enhancing the salient edges using unsharp masking where a smoothed version of the image is subtracted from the original image to obtain the unsharp mask. This mask is then added to the original image to generate the edge enhanced image. We used Gaussian smoothing to generate the unsharp mask as follows.


$$I_{EE}=I(x,y)+\left[I(x,y)-\left[\frac{1}{2\pi \sigma^{2}}e^{\frac{-u^{2}+v^{2}}{2\sigma^{2}}}*I(x,y)\right]\right]$$ (2)

where I is the input images, I_EE_ is the edge enhanced image, (*) is the convolution operation, σ is the standard deviation of the filter and was set to 0.5, x and y are the spatial coordinates of the image.

Four geometric transformations including two flips and two rotations were obtained and added to the augmented dataset to allow for a certain degree of transformation invariance. In the final dataset, each image had 8 other versions which sufficiently enlarged the dataset.

### 3.2 Deep convolutional neural network

Convolutional neural networks have emerged as powerful hierarchical architectures, capable of learning features from data automatically. They have been applied to a wide variety of applications in computer vision [20,21], natural language understanding [22], speech recognition [23], neuronal signal understanding [24], and drug discovery [25]. Their application to a field is merely limited by availability of data and its representation to these architectures for processing. A typical CNN is composed of a variety of data processing layers arranged in the form of a hierarchy, where the output of a layer becomes the input of the succeeding layer. A majority of these layers are convolutional layers which act as receptive fields for the visual data being processed. In each convolutional layer, a set of learned kernels are applied on the entire image to detect patterns at different spatial locations and generate feature maps. Pooling layers are often used after convolutional layers, which attempt to extract the most meaningful information from the set of feature maps. A common pooling strategy is to apply max pooling in which maximum activations in small neighborhoods of the image are gathered. Consequently, it reduces the dimensions of the feature maps based on the size of the local neighborhood considered for pooling. Stacks of convolutional and pooling layers are followed by fully connected layers which model higher level abstractions. In such a hierarchical setting of layers, as we go higher in the hierarchy, more abstract and semantically meaningful associations among the data are modeled.

**Fig 3:**
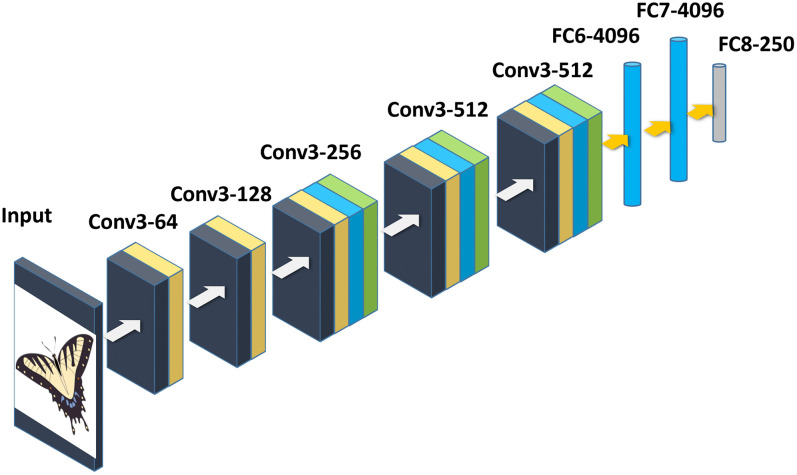
Architecture of the deep CNN for features extraction. Butterfly image from https://publicdomainvectors.org available under a CC0 license.

The CNN model we used for our experiments (shown in Fig 3) was trained by the visual geometry group (VGG) of the University of Oxford [26]. The model receives input of size 224 x 224 x 3. It has increased depth (19 layers) and used smaller convolutional kernels throughout the entire network (3 x 3 stride 1). It also used uniform pooling operations (2x2 stride 2) after each stack of convolutional layers as shown in Fig 3. The first two stacks had two convolutional layers each with 64, and 128 kernels, respectively. Two convolutional layers stacked together effectively constitute a receptive field of 5x5. The remaining three stacks consisted of four convolutional layers having 256, 512, and 512 kernels, respectively. To allow the extended depth, the input image was padded to preserve its size before each convolution operation. The two fully connected layers had 4096 neurons each. The last fully connected layer was modified according to our dataset and was set to 250 neurons, where each neuron correspond to one of the 250 classes of sketches and associated images.

### 3.3 Training CNN with augmented data

Training of a CNN is accomplished by tuning the various parameters and biases in all the layers of the model according to the input data and classification problem. It involves two stages namely forward and backward propagation phases. During the forward propagation phase, input images are forward propagated through the network with existing parameters. The loss cost is computed using the differences in predicted and ground truth labels. During the backward propagation phase, gradients of each parameter are computed using chain rules in order to adjust the parameters (weights and biases) and reduce error. These two phases are performed many times and the parameters are adjusted until the loss cost has been sufficiently reduced. We trained several models with our data and evaluated their performance for sketch classification and SBIR. Individual models similar to the architectures of AlexNet [17] and VGG-19 [26] were trained on our dataset. The models trained from scratch were able to obtain classification accuracy of 64% and 68%, respectively, which was slightly below the state-of-the-art. In order to improve the accuracy, we used the transfer learning approach where a pre-trained model is fine-tuned on the new dataset [27]. This way the classification problem is solved more effectively, thereby increasing accuracy. The final model (shown in Fig 3) had 166 million parameters. It was obtained by fine-tuning a pre-trained model (ImageNet dataset) on the augmented dataset with 250 classes. The Softmax layer of this model outputs predictions for all the classes.

In recent studies, it has been shown that transfer learning approach can enhance classification accuracy on new datasets [27]. During this approach, the classification function of pre-trained CNN model is replaced with a new classification function, and optimized to reduce classification error in the new domain. The learning rate is usually set very low (usually one-tenth of the original learning rate) so that most of the parameters and weights of the pre-trained model are only slightly modified. Consequently, the previous knowledge of the model is used to solve the new problem more efficiently. We evaluated transfer learning using the augmented dataset and consequently, a 12–15% improvement was noticed in the classification accuracy. This improvement is due to the fact that the pre-trained model has been trained on a very large dataset (ImageNet [28]) where it has learned very fine and highly discriminative features. Reusing these features significantly improves retrieval accuracy.

### 3.4 Sketch representation with deep features

**Fig 4:**
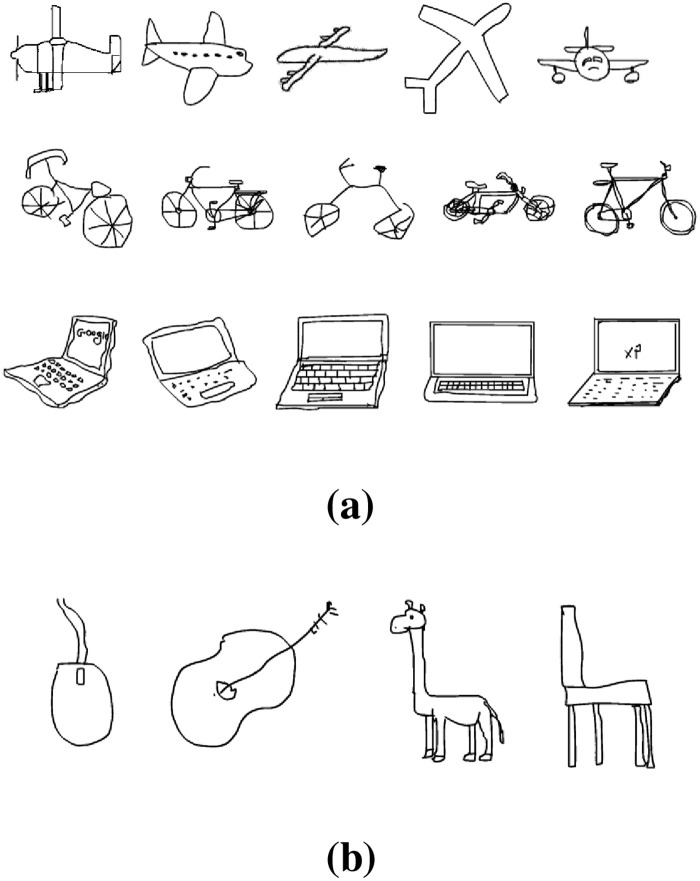
Challenges in simple sketch representation (a) visual differences in same class objects (b) inter-class similarities in sketches. Images obtained from Sketches dataset [30] republished under a CC BY license with permission from Marc Alexa, TU Berlin original copyright 2012.

**Fig 5:**
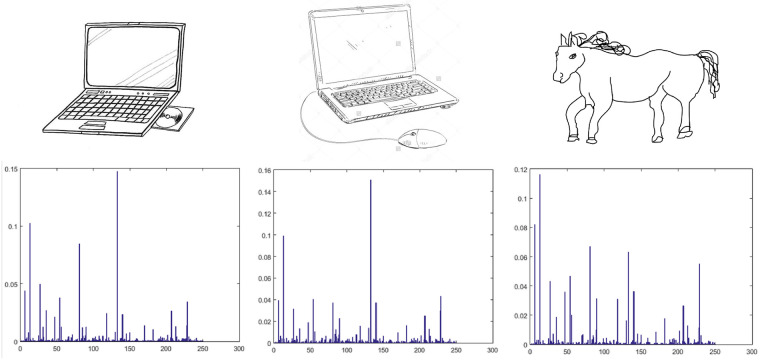
Sketch representation with deep features. Images obtained from Sketches dataset [30] republished under a CC BY license with permission from Marc Alexa, TU Berlin original copyright 2012.

The hierarchical nature of the deep CNN allows it to learn multiple levels of features from the training data. The lower layers learns relatively lower level features corresponding to edges, curves, and color blobs. Subsequent layers learn higher level features and contain more semantic features of the visual contents. Neuronal activations at various layers of the network correspond to the intermediate representation of the image. Each of these intermediate representations can be used to represent images for the task of classification or retrieval. However, it has been noticed that the higher layers in the network learn more discriminative and domain specific features [15]. Therefore they perform better than the lower layer features. We evaluated features extracted from the last three fully connected layers (FC6, FC7, and FC8) and found that the last fully connected layer (FC8) consisting of 250 neurons was the most suitable for this task. Features from this layer are discriminative and yields lower dimensional features which are favored in retrieval applications. Fig 4 shows sample sketches from the sketches dataset. It can be seen that there exist a great degree of intra-class variations (Fig 4a) as well as inter-class similarities (Fig 4b) in hand-drawn sketches which make their classification a very challenging task. Fig 4a shows five different sketches of planes, bicycles, and laptops. Sketches of computer mouse, guitar, giraffe, and chair, shown in Fig 4b exhibits inter-class similarities among the hand-drawn sketches. Features extracted for some of the sketches, shown in Fig 5 reveal that similar features are extracted from sketches belonging to similar classes, despite the differences in their visual appearances. It shows the discriminative capability of the proposed model which is key to improved retrieval performance.

### 3.5 Sketch-based image retrieval with deep features

Features extracted from the last fully connected layer of the model are used to index sketches as well as color images. When a query is submitted to the SBIR system, the same model is used to extract features from the query sketch and compared with all the images in the dataset. The full Python code used to generate the retrieval results and reproduce the plots for Figs 7, 9, and 10 is provided in S1 File. The comparison is performed by computing Euclidean distance between the query sketch and database image features. Lower the score, greater will be the similarity and vice versa. The database images are ranked according to this score in ascending order, where lower score images are retrieved at higher ranks. In the current work, we developed a software for smart devices including tablets and phones, which allow users to create sketches and partially color them before submitting them as queries to the retrieval system. The application uses deep CNN model trained using the Caffe framework [29] on the device. Though, it is relatively slower in execution due to the computational limitations of the portable devices, the performance can be significantly improved if cloud based service is used to perform the compute-intensive task of features extraction and matching. Retrieval performance of the proposed framework is presented and discussed in the subsequent section.

## Experiments and results

Sketch based image retrieval has been investigated for quite a long time. However, only limited works has been seen using deep CNN for features extraction. Two of the most relevant works to the proposed method are [18] and [9], who used convolutional neural networks to represent sketches or match sketches with images. We provide a comparison of performance with these methods and show that our method is better than both of them in terms of effectiveness and efficiency.

### 4.1 Datasets

#### TU Berlin sketches dataset [30].

This dataset is composed of 20,000 hand-drawn sketches made by non-experts. These sketches belong to 250 different categories, where each category has 80 sketches. The size of each image is 1111 x 1111. Seventy five percent of the dataset was used for training and fine-tuning the models, and the remaining data was used for testing. The test set was used as query images for searching relevant images in full color image datasets.

#### Color images dataset.

To assess the capability of deep CNN features in retrieving color images in response to partially colored sketches, we collected more than 35,000 color images from various datasets, corresponding to the 250 categories of TU Berlin sketches dataset. These images were gathered from Corel-10k dataset [31], Multi-view objects dataset [32], and Caltech256 [33].

### 4.2 Experiments design

We designed several experiments to evaluate performance of the proposed method on sketch classification and SBIR for partially colored sketches. We are tested the representation capability of CNNs for sketches with or without shading or colors. For training or fine-tuning CNNs, the training set and test sets used had no overlap in order to allow fair comparison. Furthermore, we evaluated the effectiveness of inclusion of color into SBIR for improved performance using deep features. CNN model training was accomplished on a PC running Ubuntu operating system, equipped with 64 GB RAM, Intel Core i5 CPU, and NVidia GeForce GTX TITAN X (Pascal) with 12 GB onboard memory, with Caffe deep learning framework [29]. For evaluating performance in sketch classification and retrieval based on deep features, MATLAB 2015a [34] was used. Further discussion on individual experiments and results is given in the following sections.

#### A. Sketch recognition.

**Fig 6:**
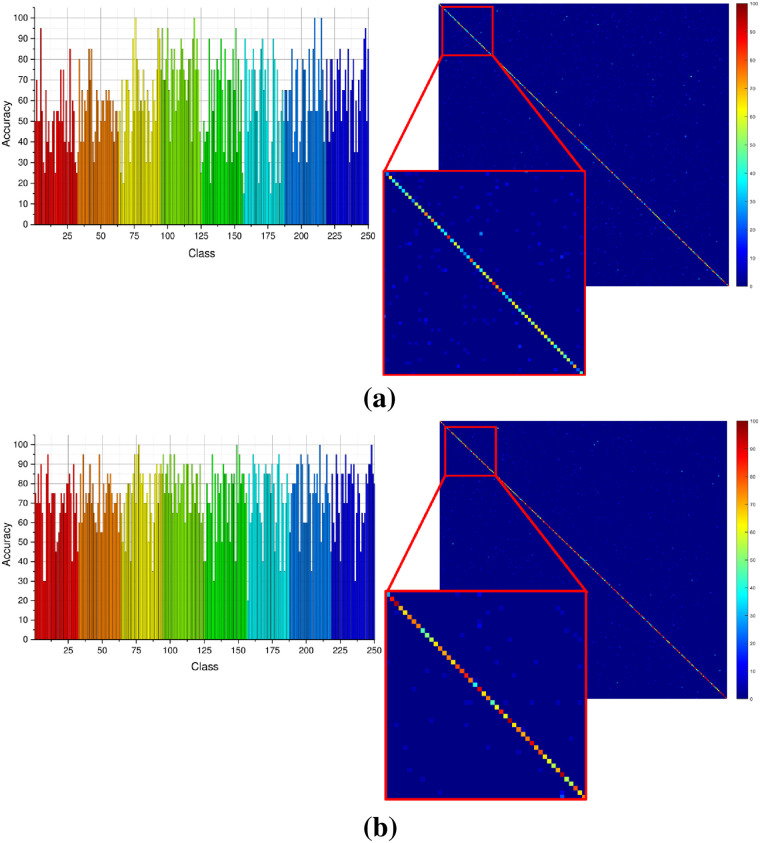
Sketch classification performance (a) without fine-tuning (b) with fine-tuned model.

**Fig 7:**
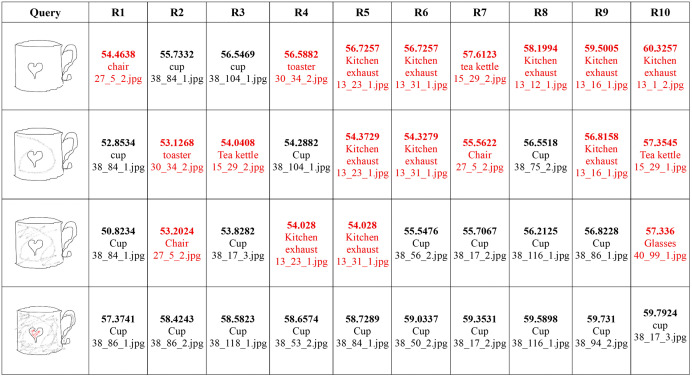
Effect of color/shade on retrieval performance. Each entry in the table contains the score (i.e., the Euclidean distance between the query image and the dataset image), the image content, and the image file name from the dataset. Query images obtained from Sketches dataset [30] republished under a CC BY license with permission from Marc Alexa, TU Berlin original copyright 2012. All retrieved images from the MVOD dataset https://www.cs.bilkent.edu.tr/~bilmdg/mvod/.

The test dataset is taken from the largest publicly available hand-drawn sketches dataset with 20,000 sketches organized into 250 categories. Twenty five percent of this dataset (5000 sketches) were combined with sketches collected from the internet to test the performance in sketch classification. Two separate experiments were performed using the selected model. During the first experiment, the model was trained using our augmented dataset for 30 epochs. Classification results for the sketch dataset with this model are provided in Fig 6. In more than 50% of the categories, the classification accuracy is above 70%. In only 20 categories, the accuracy is below 40%. In the second experiment, we used transfer learning approach to fine-tune the same model on our dataset. Experimental results shown in Fig 7 exhibit the improvement in terms of classification accuracy. Only 8 sketch categories are classified with less than 40% accuracy. Furthermore, recognition performance for most of the categories is significantly improved raising the overall accuracy from 68% to 79%.

#### B. Sketch-based retrieval.

In this experiment, we extracted features from both sketches and full color images using the model trained on augmented dataset. Images were indexed using these features. Retrieval performance of the proposed method is evaluated on a variety of sketches and edge maps. Initially we assessed the representation performance of our model for color-less sketches. In this experiment, we extracted features from the edge maps of natural images using the model. Then, randomly chosen images were used as queries to retrieve similar images from the dataset. During the experiments, relevant images were retrieved from the dataset in most cases, even if there was no color information involved during the features extraction phase. Still, the features were discriminative enough to retrieve visually similar images. It is interesting to note that the retrieved images had very similar edge maps, which lead to their retrieval at top ranks. In some of the cases, the SBIR system failed to retrieve relevant images, but when they were partially colored, their retrieval performance improved dramatically (shown in Fig 7). It showed that the introduction of colors significantly improves performance even if they are only partially applied. It also corresponds to the ability of modeling colors by the deep CNN. In order to take advantage of the modeling capabilities of CNNs, we propose to use partially colored sketches instead of simple strokes.

#### C. Effect of color on sketch-to-image retrieval.

**Fig 8:**
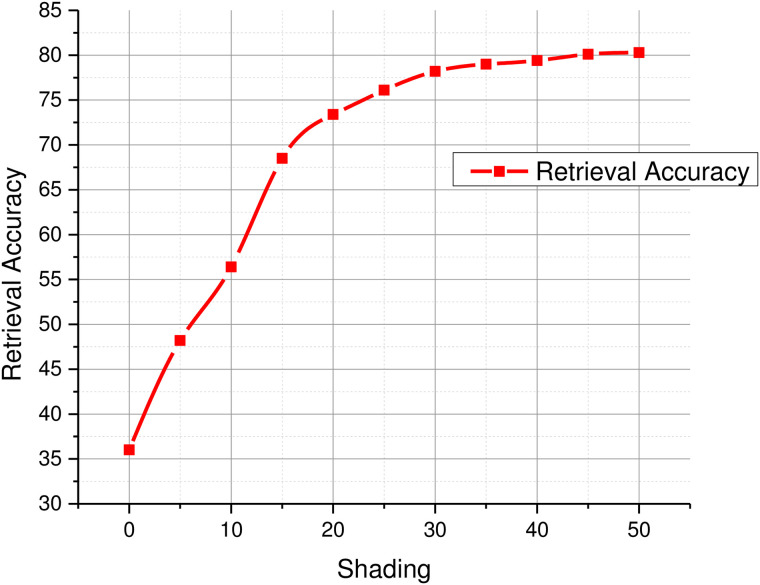
Relationship between partial shading and retrieval performance.

CNNs are powerful architectures capable of modeling visual contents including colors, textures, and shapes, along with their spatial features which lead of their semantic interpretation to a certain degree. However, sketches usually lack colors or textures which limits the discriminative power of CNNs. It has been proved in the past that color is a powerful descriptor [35–38]. In this experiment, we study the effects of colors on retrieval performance in the proposed framework. Several experiments were conducted with colorless sketches as well as their partially colored or shaded versions. Though in the past, coloring sketches was considered burdensome for the users, the convenience provided by the touch screen devices make it relatively convenient for them to sketch and apply colors to it. During the experiments, we noticed that even a single stroke of shade or color on the sketch improved retrieval performance significantly as can be seen in Fig 7. In the first image, there is no shading or colors on the sketch. The ten images shown on the right are the top-10 retrieved images. Only 3 relevant images have been retrieved out of 10 at ranks 2, 3, and 7. When a single stroke was applied to the sketch the number of relevant images increased to 5, retrieved at ranks 1, 3, 4, 8, and 10. Adding a few more strokes increased number of relevant images to 6, and further addition of the red color stroke increased the number of relevant images to 10. This experiment showed that addition of colors to sketches significantly improves their representation by deep CNNs which eventually leads to improved retrieval performance. Quantitative assessment of partial shading has also been carried out. Experimental results presented in Fig 8 report the retrieval accuracy for top 25 retrievals. Results reveal that adding only 5% shading in any sketch improves the retrieval accuracy by 12%. Similarly, increasing the amount of shading to 20% increases the retrieval accuracy to 73.4% and a 30% shade yields more than 78% retrieval accuracy. These results show that partial shading or coloring significantly improves image retrieval performance using the proposed approach.

#### D. Retrieval performance for deep features extracted from various layers.

CNNs learn multiple layers of features from the training data automatically. Neuronal activation from each of these layers can be used to represent images for image retrieval. However, the retrieval performance of the last fully connected layers are shown to outperform the early convolutional and pooling layers. Therefore, in this experiment, we evaluated the retrieval performance of various layers. Features from the last three fully connected layers were extracted to represent the database images and then retrieved with sketches having only 35–50% color. Table 1 shows the retrieval performance of various layers for both sketch classification and retrieval performance. Features extracted from FC8 showed improved performance than the other two layers.

**Table 1:** Comparison of sketch classification approaches.

Method	Classification Accuracy
SIFT-Variant+BOW+SVM [30]	56.0%
Stargraph + KNN [39]	61.5%
MKL [40]	65.8%
SIFT+FV(FMM)+SVM [41]	68.9%
Humans Recognition [30]	73.2%
Sketch-a-Net [42]	74.9%
DeepSketch [18]	77.3%
**Proposed Method (FC6-4096)**	**76.3%**
**Proposed Method (FC7-4096)**	**77.6%**
**Proposed Method (FC8-250)**	**79.1%**

#### E. Visual retrieval results for partially colored sketches.

**Fig 9:**
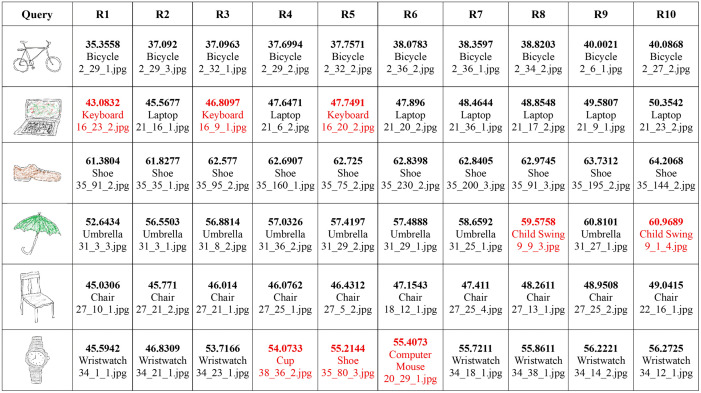
Retrieval performance in response to partially colored sketches. Each entry in the table contains the score (i.e., the Euclidean distance between the query image and the dataset image), the image content, and the image file name from the dataset. Query images obtained from Sketches dataset [30] reproduced under a CC BY license with permission from Marc Alexa, TU Berlin. All retrieved images from the MVOD dataset https://www.cs.bilkent.edu.tr/~bilmdg/mvod/.

Access to visual information can be made more convenient with the help of SBIR. Users can draw partially colored sketches of objects they are interested in, and the retrieval system would attempt to retrieve the relevant images. In this experiment, some hand-drawn sketches were partially colored and submitted as queries to the proposed SBIR system to retrieve relevant images as shown in Fig 9. For thinner shapes like bicycles, and glasses, there was no need to apply any shading or colors on the sketch and relevant (visually similar) images were retrieved with high accuracy. However, the retrieval performance for the rest of the sketches improved significantly when colors or shades were applied to them. For instance, there are viewpoint changes in laptop, umbrella, and chair, yet the proposed system was able to retrieve them. Although, some irrelevant images have been retrieved for laptop, umbrella, chair, and watch, retrieval performance got improved as more color was added to the sketch.

**Fig 10:**
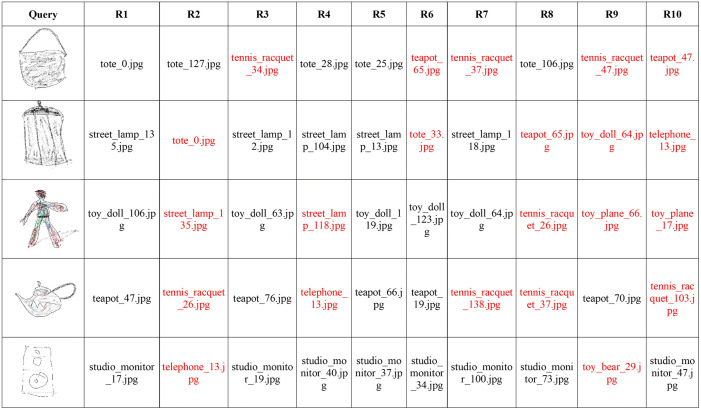
Retrieval performance on other categories. Against each query image, the top ten images were retrieved from the dataset. Query images obtained from Sketches dataset [30] reproduced under a CC BY license with permission from Marc Alexa, TU Berlin. All retrieved images from the MVOD dataset https://www.cs.bilkent.edu.tr/~bilmdg/mvod/.

In addition, we also experimented with sketches and images other than the ones used in the training or validation. In some cases, there exist less ambiguity between the sketch and corresponding images without any shades or colors such as a tennis racquet, bicycle, and glasses, etc. But for others, the ambiguity can be significantly reduced by adding some shades. Results in Fig 10 suggest that the proposed method can perform well with a huge variety of images other than the ones used during training. The first image in each row is the query sketch and the remaining are top 10 retrieved images from a large dataset of images. Though some incorrect images have been retrieved within the top 10 results, the relevant images have been retrieved at higher ranks. These results can be further improved if more colors or shades are added to them. The results validate the effectiveness of proposed approach in real world scenarios.

## Conclusion and future work

In this paper, we present a method for sketch-based image retrieval system which uses partially colored hand-drawn sketches to allow access to visual data in educational applications. We slightly modified a deep CNN pre-trained on ImageNet dataset and fine-tuned it on augmented dataset, composed of sketches, color images, edge maps, de-colorized, and de-texturized images. The images belong to 250 categories and consisted of very challenging sketches. The model’s capabilities were extensively evaluated for representing hand-drawn sketches for image retrieval applications. The main aim was to allow users to supply hand-drawn partially color sketches as queries and access full color images from the dataset. We observed that the model is able to retrieve thin shapes like eyeglasses and bicycles using rough colorless sketches very effectively. The rest of the objects were retrieved with relatively less accuracy. However, it was interesting to note that the introduction of colors to sketches significantly improved the retrieval performance based on the degree of color or shade applied to the sketch. Even a single stroke of color or shade would improve retrieval performance for almost any sketch, and this improvement was directly related to the amount of color or shade applied to it.

Traditionally, it was believed that drawing full color sketches in SBIR systems was very difficult for end users. However, we believe, that the touch screen devices have made it convenient for users to quickly draw and color sketches using on screen controls and submit their sketches as queries. Rather than devising relevant feedback strategies to refine search results, it is far more convenient and efficient to attempt at retrieval with a little bit effort in preparing the queries. The results show that there is promise in the proposed approach and further improvements can be achieved if more work is done along these lines.

## Supporting information

S1 File Python code used to generate the retrieval results and reproduce the plots for Figs 7, 9, and 10. (ZIP)

S2 File Data underlying Fig 8. (ZIP)
